# Natural Environment Suitability of China and Its Relationship with Population Distributions

**DOI:** 10.3390/ijerph6123025

**Published:** 2009-12-01

**Authors:** Xiaohuan Yang, Hanqing Ma

**Affiliations:** 1 State Key Lab of Resources and Environmental Information System, Institute of Geographical Sciences & Natural Resources Research, Chinese Academy of Sciences (CAS), Beijing 100101, China; E-Mail: mahq.07s@igsnrr.ac.cn; 2 Graduate School of the Chinese Academy of Sciences, Beijing 100049, China

**Keywords:** natural environment, population, GDP, spatial distribution, GIS, NESI

## Abstract

The natural environment factor is one of the main indexes for evaluating human habitats, sustained economic growth and ecological health status. Based on Geographic Information System (GIS) technology and an analytic hierarchy process method, this article presents the construction of the Natural Environment Suitability Index (NESI) model of China by using natural environment data including climate, hydrology, surface configuration and ecological conditions. The NESI value is calculated in grids of 1 km by 1 km through ArcGIS. The spatial regularity of NESI is analyzed according to its spatial distribution and proportional structure. The relationship of NESI with population distribution and economic growth is also discussed by analyzing NESI results with population distribution data and GDP data in 1 km by 1 km grids. The study shows that: (1) the value of NESI is higher in the East and lower in the West in China; The best natural environment area is the Yangtze River Delta region and the worst are the northwest of Tibet and southwest of Xinjiang. (2) There is a close correlation among natural environment, population distribution and economic growth; the best natural environment area, the Yangtze River Delta region, is also the region with higher population density and richer economy. The worst natural environment areas, Northwest and Tibetan Plateau, are also regions with lower population density and poorer economies.

## 1. Introduction

The natural environment where people produce, multiply, live and develop can supply all kinds of natural resources and energy, but it is also being continually transformed by human activities. Many scholars have studied the characteristics of the natural environment and its spatial differences to achieve a natural and ecological division [1–14]. The diversity of the natural environment and unbalanced distribution of its elements have been one of the basic reasons resulting in regional economic development differences [15]. The natural environment on national scales includes topography, temperature, water, biology, soil and other factors [10,16]. Among these factors, climate conditions (such as temperature, precipitation and sunlight) and landform conditions (such as topographic slope, sea level elevation and undulating range of land surface) directly influence human life and economic development.

With the development of remote sensing and GIS technologies, it is possible to acquire time series data of natural environment factors for evaluating human habitats, sustained economic growth and ecological health status. The ecosystem assessment, to some extent, is the evaluation of the natural environment. Examples that involve factors of natural environment as evaluation indexes include the Global Environment Outlook program [17,18] of the United Nations Environment Program (UNEP), the Heinz Center evaluation of America’s ecosystems [19], the Millennium Ecosystem Assessment [20,21] by the United Nation, and the Ecological Indicators for the Nation [22] by the US National Research Council. Jing has used natural environment and social economic data to evaluate the sustained urban development in Jing-Jin-Ji region of China [23]. Li has evaluated the influence of natural environment on the difference in regional development [15].

Temperature, precipitation and sunlight conditions are the main factors for agricultural production. Some studies have shown that the main weather factors for food production in Heilongjiang Province of China are average temperature in June, precipitation in September and hours of sunshine in May and June [24]. The spatial differences in the natural environment are a key factor for population distribution. Feng has analyzed the influence of undulating range of land surface to population distribution in China [25]. Dong has shown that there is a certain correlation between natural factors and population distribution [26].

To quantitatively analyze the natural environment in China and expose its spatial distribution regularity, in this study, remote sensing and GIS technologies are used to acquire quantitative information on the natural environment, and analytic hierarchy process method is employed to construct the NESI model. NESI values in grids of 1 km by 1 km are calculated in ArcGIS. The distribution regularity of NESI and its relationship with population distribution and economic growth are analyzed in detail. The main purpose of this article is exposing the regularity of natural environment in China and its relationship with population distribution.

## 2. Research Method

### 2.1. Natural Environment Evaluation

#### 2.1.1. Factor Selection

Since natural environment factors are diverse, factor selection is an important process in evaluating the natural environment scientifically and systematically. Huang has studied the natural environment of China based on topography, temperature, water, biology, soil and other factors [10,16]. Gao has concluded that the important natural factors related to human life are hydrothermal conditions [27]. Temperature, precipitation and sunlight conditions are also the main factors for agricultural production [24]. On the basis of those results and our research objective, considering the availability of data acquisition, nine parameters in four factors are selected to form the evaluating index system (Table 1).

#### 2.1.2. Data Acquisition and Processing

According to the evaluation criteria system mentioned above, the input data for this study include:

1. DEM data (including elevation and slope), feature types and transportation data were supplied by State Bureau of Surveying and Cartography (SBSC).
2. Meteorological data, including annual average precipitation, accumulated air temperature, wetness index and sunlight, were derived from National Resources and Environmental Database presented by Resources and Environmental Scientific Data Center (RESDC), Chinese Academy of Sciences (CAS). The accumulated air temperature refers to the annual daily mean air temperature above 0 °C. Wetness index is defined as actual evapotranspiration dividing by potential evapotranspiration, which was calculated from meteorological data annually.
3. Net primary production (NPP) data were also derived from RESDC/CAS.
4. River density data, which refers to the length of the river per unit area within certain statistical unit, were derived from land use data for 2005. The land use data were obtained from Landsat TM data through human-computer interactive interpretation by RESDC.

Before further processing, all of the input data were re-sampled into a raster dataset with 1 km spatial resolution. Meanwhile, the data were transformed to the same coordinate system, *i.e.*, Albers Equal Area projection system with original longitude 105 °E, double standard parallel of 27 °N and 45 °N, Beijing 1954 geodetic datum and Krassovsky ellipsoid.

#### 2.1.3. Evaluating Model Construction

As one of the main indexes to evaluate the natural environment in China, the Natural Environment Suitability Index (NESI) would serve for evaluating the national natural environment on large scales. In this study, the Analytic Hierarchy Process (AHP) method is used to determine the weight coefficients of all indexes. The Analytic Hierarchy Process method, put forward by American Operational Researcher T. L. Saaty in the 1970s, has been widely applied to natural environment studies [27–29]. In this method, decision makers divide complex issues into a number of levels and factors, and then obtain the weight coefficients of different plans by a brief comparison and calculation among the factors. In this study, weather, hydrological, topographical and ecological conditions are level-1 indexes. Selected factors of each level-1 index are level-2 indexes. According to the relative importance of various levels, the suitable decision matrix is constructed; Sorted weight coefficients in a single level can be determined by solving the eigenvalue of every judgement matrix (Table 2).

Standardized processing must be done because the various indexes could not be compared by different units. Therefore, each factor is classified into 6 grades from the original values (Table 3).

The equation of the NESI model is shown as:

(1) $$\text{NESI}\;=\;\sum {\text{A}}_{\text{i}}{\text{W}}_{\text{i}}^{*}{\text{W}}_{\text{j}}$$

In the formula, A_i_ denotes the standard value of level-2 index, which is 0, 2, …, 10; W_i_ denotes the weight coefficients of level-2 index; W_j_ denotes the weight coefficients of level-1 index. When the value of NESI is bigger, the natural environment is better.

### 2.2. Spatial Population and Economy Data Acquisition

#### 2.2.1. Spatial Population Data Acquisition

**Figure 1.**
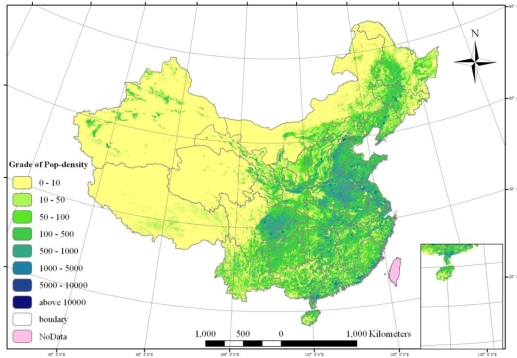
Population Density Distribution of China in 2003.

According to the division of population distribution [30], several steps are used to calculate population density distribution: (1) construct a multiple correlation model between statistical population and land use types; (2) calculate the population distribution coefficients of every land use type; (3) obtain the spatial population distribution of 1 km by 1 km girds through GIS; and (4) rectify the results with DEM data. The population density distribution of China is shown in Figure 1. Specific calculation methods can be found in the literature [31,32].

#### 2.2.2. Spatial Economy Data Acquisition

**Figure 2.**
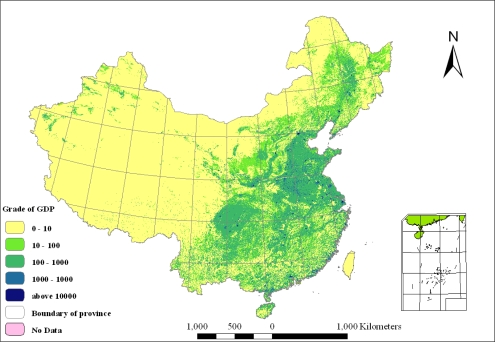
GDP Density Distribution of China in 2003.

Gross Domestic Product (GDP) is one of the most important indexes to reflect the overall economic growth of a certain country or area. Using land use distribution of 1:100,000 scale based on remote sensing data in 2000, and comprehensively analyzing the mutual spatial regularity between GDP and land use statement formed by human activities, evaluating model of key factors which impact economic growth is accomplished. Through the spatial correlation between land use types and three major industries of the GDP, a spatial model base of correlation between GDP and land use types on a county level in 2003 is done to fulfill the quantitatively spatial simulation of social and economic data in 1 km by 1 km grids (Figure 2). Specific calculation methods can be found in the literature [33,34].

## 3. Results

### 3.1. Spatial Pattern of NESI

**Figure 3.**
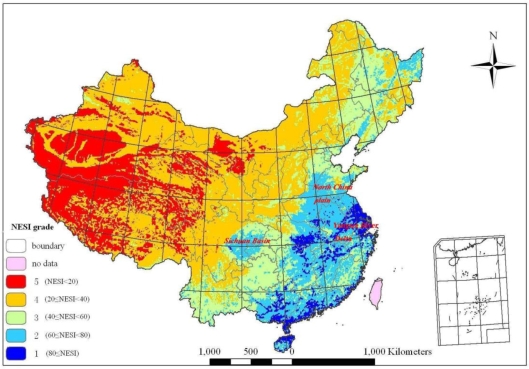
Distribution of NESI.

According to the NESI model, the natural environment suitable index of China is calculated for 1 km × 1 km grids. According to the value of natural environment indexes, the natural environment in China have been divided into five grades: fifth-grade (NESI < 20), fourth-grade (20 ≤ NESI < 40), third-grade (40 ≤ NESI < 60), second-grade (60 ≤ NESI < 80), and first-grade (80 ≤ NESI) [27]. The fifth-grade is the worst environment area, while the first-grade is the best (shown in Figure 3). The area percent of these five grades are respectively 2.93%, 11.91%, 20.44%, 43.34% and 21.37%.

It is shown in Figure 3 that NESI presents a declining trend from the southeast to the northwest of China. The highest value of NESI distributes on the Yangtze River Delta region and middle reach area of the Yangtze River. The second highest value of NESI distributes on the Sichuan Basin and North China Plain. The lowest value of NESI distributes on the northwest of Tibetan Plateau and southwest of Xinjiang province.

### 3.2. Spatial Pattern of Population

Figure 1 shows that the distribution pattern of population in China in 2003 is still dense in the east and sparse in the west. Population density presents a declining trend from the southeast to the northwest of China.

The whole nation is divided into two parts by using the Hu-Huanyong Line: the northwest and the southeast [36]. The northwest part has an average population density of 14 people/km^2^, with a large area where no people live and a few cities with high population density. The northwest part covers 57.2% of the total area of China, with only 6% of the national population. The southeast part has an average population density of 301 people/km^2^, with high population density areas mainly distributed in the North China Plain, Sichuan Basin, middle and lower reaches of Yangtze River and large cities with higher population density. The southeast part covers 42.7% of the total area of China, but 94% of the national population.

### 3.3. Spatial Pattern of GDP

Figure 2 shows that the GDP density presents a declining trend from the southeast to the northwest of China. To match the population analysis, the whole nation is also divided into the same two parts by the Hu-Huanyong Line. The GDP in the northwest is 3.53% of the whole nation, while the GDP in the southeast is 96.58%. The difference of economic growth is huge between the east and the west. Except some large cities, the GDP of most areas in the west are much lower than the middle and east area. The 1 km grid can show the GDP value and exact location on every 1 km by 1 km area, so it can further show the rural-urban differences. It is shown from Figure 2 that the area with high GDP value is distributed in large and middle cities and around areas, such as municipalities, capital cities and prefecture-level cities.

## 4. Analysis and Discussion

By analyzing the latitude, longitude and sea level elevation of the NESI in China, we have quantitatively calculated correlations between the NESI and population distribution in different areas and exposed the influence of NESI on population distribution on a 1 km by 1 km grid scale.

### 4.1. Population Distribution and GDP in Different NESI Areas

By analyzing the distribution regularity of the natural environment in China, overlay analysis between grading results and grid data of population or GDP has been made for the purpose of revealing the relationship between natural environment conditions and population distribution or economic growth on a regional scale. The results show that the natural environment condition is a major factor for population distribution, and that the population distribution obviously concentrates on better natural environment areas (Table 4).

More details are provided below:

1. The fifth-grade of natural environment. The NESI values are between 0 and 20. The topography and climate conditions are so bad that people could not easily live in such arctic-alpine and arid areas. The area of this category is 201.42 × 10^4^ km^2^, or 21.37% of the total area. The population is 2.27 million, about 0.18% of the total population. The GDP is 6.38 billion RMB, about 0.08% of the total GDP. This category mainly distributes in the Junggar Basin, Tarim Basin, Alxa Plateau of the northwest arid regions, the Northern Tibet Plateau, Qaidam Basin, south of Qinghai and southeast of Tibet. These regions have few people owing to the restraint of topography, vegetation, water, climate and other factors. The economic growth of these regions is very backward.
2. The fourth-grade of natural environment. The NESI values are between 20 and 40. The topography and climate conditions are better than those of the fifth-grade. The area is 408.50 × 10^4^ km^2^, 43.34% of the total area. The population is 161 million, 12.59% of the total population. The GDP is 583.14 billion RMB, 7.56% of the total GDP. It is a transitional region between regions suitable for human living and those not suitable for human living, like in the southeast edge of Tibetan Plateau, Junggar Basin, northern Loess Plateau, Daxinanling and Xiaoxinanling area, with a population density of 18 people/km^2^. The economy is obviously developed only in the main cities.
3. The third-grade natural environment. The NESI values are between 40 and 60. The natural condition has improved a lot. The area is 192.65 × 10^4^ km^2^, about 20.44% of the total area. The population is 406 million, 31.67% of the total population. The GDP is 2267.98 billion RMB, about 29.39% of the total GDP. It mainly distributes in the Northeast Plain, northern North China Plain, southern Loess Plateau, northern Yunnan-Guizhou Plateau, and southern valley of Tibet. The population density is 211 people/km^2^, slightly higher than the national average. The economic growth is much improved.
4. The second-grade natural environment. The NESI values are between 60 and 80. The natural conditions are fine and the hydrothermal resources are abundant. The area is 112.65 × 10^4^ km^2^, about 11.91% of the total area. The population is 518 million, about 40.36% of the total population. The GDP is 3003.78 billion RMB, 38.93% of the total GDP. It mainly distributes in the eastern Yunnan-Guizhou Plateau, Sichuan Basin, Middle and Lower Yangtze Valley Plain, Guanzhong Basin and southern North China Plain. The population density is 462 people/km^2^, the most populated area in China, also the highest GDP area in China.
5. The first-grade natural environment. The NESI values are over 80. The natural conditions are very good and climate is suitable. The area is 27.62 × 10^4^ km^2^, about 2.93% of the total area. The population is 195 million, 15.2% of the total population. The GDP is 1,854.43 billion RMB, 24.03% of the total GDP. It mainly distributes in the Middle and Lower Yangtze Valley Plain, Southeast Area and part of Sichuan Basin. The population density is 707 people/km^2^, the highest among the five categories. The economy is the most developed.

The above results show that the first-grade and second-grade natural environments only have 14.84% of the total area, but 55.6% of the population and 62.9% of GDP. On the other hand, the fourth-grade and fifth-grade natural environments have 64.72% of the total area, but population is only 12.8% and GDP is only 14.6%. These results suggest that the quality of natural environment is related to population distribution and economic growth.

### 4.2. NESI Features in Different Population Density Areas

According to different values of population density, China is divided into eight grades of population density (shown in Figure 1 and Table 5). Based on the grades of population density, proportion of the population and proportion of area are used to further discuss the geographic characters and spatial patterns of the population distribution.

The results show that the population distribution in China is very uneven. The regions of more than 500 people/km^2^ contain 67.6% of the total population but only 6.6% of the total area, which further demonstrates that the population distribution in China is highly congregated. Conversely, the regions with less than 10 people/km^2^ have only 0.12% of the total population but 66.54% of the total area, mostly uninhabited.

In the regions with population density less than 10 people/km^2^, 31.96% distributes in the fifth-grade natural environment and 52.68% in the fourth-grade environment. The regions with population density between 10 and 50 people/km^2^ are mainly distributed in the fourth-grade environment. The regions with population density between 50 and 500 people/km^2^ mainly correspond to the third-grade environment. The regions with population density over 500 people/km^2^ are mainly distributed in the second-grade environment.

### 4.3. NESI Features in Different GDP Density Areas

According to the different values of GDP density, China is divided into five grades of GDP density (shown in Figure 2 and Table 6). Based on the grades of GDP density, the proportion of the GDP and proportion of area are used to further discuss the geographic characters and spatial patterns of the GDP distribution.

The results show that the GDP distribution in China is very uneven. The regions with more than 100 million RMB/km^2^ have 39.24% of the total GDP but only 0.24% of the total area, which further demonstrates that the GDP distribution in China is highly congregated. Conversely, the regions with less than 100 thousand RMB/km^2^ have only 0.99% of the total GDP but 74.11% of the total area, mostly uninhabited.

In the regions with GDP density less than 100 thousand RMB/km^2^, 28.83% is distributed in the fifth-grade natural environment and 52.09% in the fourth-grade environment. The regions with GDP density between 100 and 1000 thousand RMB/km^2^ are mainly distributed in the third-grade environment. The other three GDP densities are mainly distributed in the second-grade environment. The regions more than 100 million RMB/km^2^ are mainly in cities, so it does not show an obvious correlation with natural environment. However, as a whole, GDP has some relationship with natural environment but it is not very close. For economic growth is impacted by other factors such as policies, the correlation is not as close as for the population.

### 4.4. NESI, Population and GDP Statistics based on Various Provinces

Based on the NESI grades, population and GDP density of the provinces, the average NESI value, population and GDP density of each province is calculated and all 31 provinces are sorted by their NESI values in Table 7.

By comparing the sequence of NESI and changing trends of population density in Table 7, the evaluation of the Natural Environment Suitability Index of China in Figure 3, the population density distribution of China in Figure 1 and the GDP density distribution of China in Figure 2, we can conclude that the best natural environments in China are Shanghai, Jiangsu, Hainan, Anhui and Guangdong, all in the east and Yangtze River Delta regions. The worst natural environments are Tibet, Qinghai, Xinjiang and Gansu, all in the arctic-alpine and arid regions. Meanwhile, the best natural environment areas are also the highest population and GDP density regions in China. So, the natural environment has directly decided the potential of regional development.

**Figure 4.**
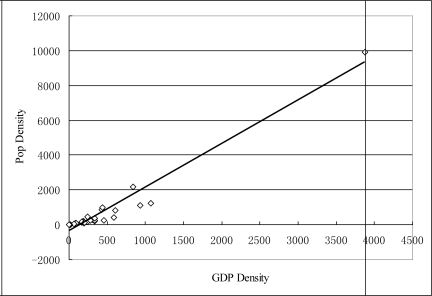
The correlation between population density and GDP in province level.

According to the average population and GDP density in each province (Table 7), correlation analysis is made under SPSS and it shows that there is close correlation between population density and GDP density. The correlation coefficient is 0.91 (Figure 4). So, the quality of natural environment has obvious correlation with population count, population density and economic growth.

## 5. Conclusions

Based on GIS technology, this article chooses climate, hydrology, topography and ecology factors as environment indexes, establishes an evaluation model and standard of natural environment, quantitatively exposes the quality of natural environment in each area and its relationship with population distributions.

The Natural Environment Suitability Indexes of China demonstrate a declining trend from the southeast to the northwest. The NESI have a close correlation with population density and reflect the quality of natural environment and suitability for human habitation.

The best natural environment areas in China are distributed in the Southeast, Sichuan Basin, and middle and lower reaches of Yangtze River. These places not only have the best natural resources, but also the highest population density, GDP density and land utilization level.

The Natural Environment Suitability Index has a direct correlation with population and population density. The population density depends on the quality of local natural environment. Meanwhile, the natural environment indirectly impacts the GDP density.

This study shows that the natural environment evaluation model based on NESI can better reflect the quality of the natural environment and suitability of people living in various regions, and provide quantitative measures of spatial regularity of the natural environment in China. In addition, the pattern and regional difference of the natural environment in China can be better represented by NESI. NESI can serve for ecological environment protection and evaluation, agricultural development planning, and other relevant applications.

## Figures and Tables

**Table 1.** Main factors and parameters for natural environment evaluation.

Factors	Parameters	Scale	Time	Data sources
1 weather & climate	1.1 accumulated temperature	1 km		
	1.2 hours of sunshine	1 km	annual average of 1981–2005	RESDC
	1.3 humidity index	1 km		
2 hydrology	2.1 annual precipitation	1 km		
	2.2 drainage density	1:100,000	2005	RESDC/CAS
3 topography	3.1 elevation (m)	1:2,500,000	2000	
	3.2 slope	1:2,500,000	2000	SBSC
	3.3 feature type	1:40,000,000	2000	
4 ground vegetation	4.1 net primary production (NPP)	1 km	2005	RESDC/CAS

**Table 2.** The evaluated indexes system and weight coefficients of the environmental background.

Level-1 index		Level-2 index	
Index	Weight	Index	Weight
1 Climate condition	0.20	1.1 Accumulated temperature	0.50
		1.2 Hours of sunshine (h)	0.15
		1.3 Wetness index	0.35
2 Hydrology condition	0.31	2.1 Precipitation (mm)	0.65
		2.2 River density	0.35
3 Topography condition	0.37	3.1 Elevation (m)	0.25
		3.2 Slope (°)	0.40
		3.3 Feature types	0.35
4 Ecology condition	0.12	4.1 NPP (g/m^2^.y)	1

**Table 3.** Data standardization.

Factors	Parameters	Standardized relative value					
		0	2	4	6	8	10
1 Climate condition	1.1 Accumulated temperature (>0°C)	<500	500–1k	1k–2k	2k–5k	5k–7k	>7k
	1.2 Hours of sunshine	<1	1–2	2–3	3–4	4–5	>5
	1.3 Wetness index	<0.05	0.05–0.15	0.15–0.3	0.3–0.45	0.45–0.65	>0.65
2 Hydrology condition	2.1 Precipitation	<100	100–200	200–400	400–800	800–1000	>1000
	2.2 River density	<10	10~30	30–60	60–100	100–150	>150
3 Topography condition	3.1 Elevation	>25	15–25	8–15	5–8	2–5	<2
	3.2 Slope	>5k	2k–5k	1k–2k	500–1k	200–500	<200
	3.3 Feature types	Sand hill, glacier	Abrupt mountain	plateau	Hills	mesa	plain and all others types
4 Ecology condition	4.1 NPP	<50	50–100	100–200	200–400	400–700	>700

**Table 4.** Area, population and GDP statistics based on the natural environment suitability of China.

NESI Grade	Land		Population			GDP		
	Area (10^4^ km^2^)	Percent (%)	Number (million people)	Percent (%)	Population Density (People/ km^2^)	Value (10^8^ RMB)	Percent (%)	GDP Density (10^4^ RMB/ km^2^)
5th	201.42	21.37	2.27	0.18	1	63.82	0.08	0.32
4th	408.50	43.34	161.76	12.59	40	5831.44	7.56	14.30
3rd	192.65	20.44	406.95	31.67	211	22679.80	29.39	118.46
2nd	112.25	11.91	518.66	40.36	462	30037.80	38.93	269.58
1st	27.62	2.93	195.40	15.21	707	18544.30	24.03	689.26

**Table 5.** The distribution of population density and natural environment.

Population density (People/ km^2^)	The area percent of various natural environment					The percent of total (%)	
	5^th^ grade	4^th^ grade	3^rd^ grade	2^nd^ grade	1^st^ grade	Area	Population
0–10	31.96	**52.68**	11.36	3.43	0.57	66.54	0.12
10–50	0.36	**42.71**	39.59	15.43	1.91	6.50	1.369
50–100	0.42	35.87	**42.81**	17.98	2.93	5.21	2.788
100–500	0.34	19.94	**41.60**	29.44	8.67	15.20	28.078
500–1000	0.13	8.84	27.15	**50.72**	13.16	4.78	23.773
1000–5000	0.13	11.47	24.47	**46.12**	17.80	1.47	18.929
5000–10000	0.17	9.41	32.40	**37.09**	20.92	0.18	9.248
>10000	0.00	7.48	33.05	**38.70**	20.77	0.13	15.703

Note: The number in bold type denotes the largest proportion of NESI grade in each level of population density.

**Table 6.** The distribution of GDP density and natural environment.

GDP density (10^4^ RMB/ km^2^)	The area percent of various natural environment					The percent of total (%)	
	5^th^ grade	4^th^ grade	3^rd^ grade	2^nd^ grade	1^st^ grade	Area	GDP
0–10	28.83	**52.09**	14.84	3.87	0.35	74.11	0.99
10–100	0.36	22.33	**39.73**	30.41	7.17	17.35	6.97
100–1000	0.09	11.69	29.52	**44.33**	14.36	7.23	26.56
1000–10000	0.08	7.18	26.88	**44.55**	24.31	1.07	26.24
>10000	0.04	5.0	30.83	**34.86**	29.27	0.24	39.24

**Table 7.** The province sequence according to the NESI of China (Not including Taiwan, Hong Kong and Macao).

Province	ranking	NESI	Population density pople/ Km^2^	GDP density 10^4^ RBM/ Km^2^	Province	ranking	NESI	Population density pople/ Km^2^	GDP density 10^4^RBM/Km^2^
Shanghai	1	83.13	3875	9923.10	Liaoning	17	60.5	280	261.09
Jiangsu	2	79.07	1076	1220.13	Heilongjiang	18	60.02	84	96.24
Hainan	3	78.69	184	205.43	Jilin	19	59.82	176	160.54
Anhui	4	76.24	454	260.80	Beijing	20	57.93	843	2180.64
Guangdong	5	75.58	427	871.37	Hebei	21	57.36	338	391.97
Jiangxi	6	73.11	239	153.46	Yunnan	22	56.47	97	53.31
Guangxi	7	72.09	193	113.35	Sichuan	23	49.8	169	117.51
Zhejiang	8	71.7	435	984.61	Shanxi	24	49.79	166	166.45
Hunan	9	71.44	340	208.23	Shaanxi	25	49.31	204	114.04
Hubei	10	69.44	311	294.45	Inner Mongolia	26	45.93	19	19.73
Fujian	11	68.52	244	451.41	Ningxia	27	43.86	96	74.87
Henan	12	68.36	590	401.14	Gansu	28	38.28	67	35.05
Shandong	13	66.27	607	833.68	Xinjiang	29	31	9	7.31
Tianjin	14	65.58	929	1126.77	Qinghai	30	30.25	6	4.27
Chongqing	15	64.14	333	261.43	Tibet	31	27.87	2	0.73
Guizhou	16	61.24	193	86.14					

## References

[1] Herberson AJ The Major Natural Region: an Essay in Systematic Geography Geogr. J 1905 25 300 312

[2] Dokuchaev VV On the Theory of Natural Zones. 1899 Sochineniya (Collected Works) Leningrad Moskow, USSR 1951

[3] Bailey RG Explanatory Supplement to Eco-regions Map of the Continents Environ. Conserv 1989 16 307 315

[4] Rowe JS Sheard JW Ecological Land Classification: a Survey Approach Environ. Manage 1981 5 451 464

[5] Bailey RG Delineation of Ecosystem Region Environ. Manage 1983 7 365 373

[6] Denton SR Barnes BV An Ecological Climatic Classification of Michigan: a Quantitative Approach Forest Sci 1988 34 119 138

[7] Klijn F Helias A A Hierarchical Approach to Ecosystem and its Implications for Ecological Land Classification Landscape Ecol 1994 9 89 104

[8] Host GE Polzer PL Mladenoff DJ White MA Crow TR A Quantitative Approach to Developing Regional Ecosystem Classifications Ecol. Appli 1996 6 608 618

[9] Fu B Chen L Liu G The Objectives Tasks and Characteristics of China Ecological Regionalization Acta Eco. Sinica 1999 19 591 596

[10] Huang B A Draft of Comprehensive Natural Zoning of China Sci. Aviso 1959 18 594 602

[11] Ren M Yang R Issues on the Nature Regionalization of China Acta Geogr. Sinica 1961 27 66 74

[12] Fu B Liu G Chen L Ma K Li J Scheme of Ecological Regionalization in China Acta Ecol. Sinica 2001 21 1 6

[13] Zheng D A Study on the Eco-geographic Regional System of China Global Ecological Zoning Workshop Cambridge, UK 28–30 7 1999 12

[14] Yang Q Zheng D Wu S Research on the Eco-geographic Regional System in China Prog. Nat. Sci 2002 12 287 291

[15] Li G Guo Z Methods to Evaluate the Impacts of Physio-geographical Pattern on the Spatio-Temporal Differentiation of Regional Development Geogr. Res 2007 26 1 10

[16] Yang Q Wu S Zheng D A Retrospect and Prospect of Researches on Regional Physio-geographical System Geogr. Res 2002 21 407 417

[17] UNEP (United Nations Environment Program) Global Environment Outlook 3: Past, Present and Future Perspectives Earth Scan Publications Ltd London, UK 2002

[18] UNEP (United Nations Environment Program) Global Environment Outlook 4: Environment for Development Progress Press Ltd Valletta, Malta 2007

[19] The Heinz Center The State of the Nation's Ecosystems: Measuring the Lands, Waters, and Living Resources of the United States Cambridge University Press New York, NY, USA 2002

[20] Millennium Ecosystem Assessment Ecosystems and Human Wellbeing: A Framework for Assessment Island Press Washington, DC, USA 2003

[21] Millennium Ecosystem Assessment Ecosystems and Human Wel1 being Synthesis Island Press Washington, DC, USA 2005

[22] NRC (National Research Council) Ecological Indicators for the Nation National Academies Press Washington, DC, USA 2000

[23] Dong J Zhuang D Xu X Ying L Integrated Evaluation of Urban Development Suitability Based on Remote Sensing and GIS Technique–A Case Study in Jingjinji Area, China Sensors 2008 8 5975 5986 10.3390/s8095975 PMC3705542 27873852

[24] Wang P Na J Analysis of Yield Components and Meteorological Factors Affecting Yields in Heilongjiang Province Meteorol. Sci. Tech 2008 36 449 452

[25] Feng Z Tang Y Yang Y Zhang D Relief Degree of Land Surface and Its Influence on Population Distribution in China J. Geogr. Sci 2008 18 237 246

[26] Dong C Liu J Zhao R Wang G A Discussion on Correlation of Geographical Parameter with Spatial Population Distribution Remote Sens. Inform 2002 4 61 64

[27] Gao Z Liu J Zhuang D The Relations Analysis between Ecological Environmental Quality of Chinese Land Resources and Population J. Remote Sens 1999 3 66 70

[28] Saaty TL Decision-making with the AHP: Why Is the Principle Eigenvector Necessary Eur. J. Oper. Res 2003 145 85 91

[29] Xu JH The Mathematical Methods in Model Geography Higher Educational Press Beijing, P.R. China 1994 10 120

[30] Yang X Wang N Jiang D Xiong L Liu H Regionalization of Population Distribution based on Spatial Analysis J. Geogr. Sci 2002 57 76 81

[31] Yang X Huang Y Dong P Jiang D Liu H An Updating System for the Gridded Population Database of China Based on Remote Sensing, GIS and Spatial Database Technologies Sensors 2009 9 1128 1140 10.3390/s90201128 PMC3280851 22399959

[32] Yang X Jiang D Wang N Liu H Method of Pixelizing Population Data J. Geogr. Sci 2002 57 70 75

[33] Liu H Jiang D Yang X Luo C Spatialization Approach to 1km Grid GDP Supported by Remote Sensing J. Geo-inform. Sci 2005 7 120 123

[34] Yi L Xiong L Yang X Method of Pixelizing GDP Data Based on the GIS J. Gansu Sci 2006 18 54 58

